# Novel Detection of Insecticide Resistance Related P450 Genes and Transcriptome Analysis of the Hemimetabolous Pest *Erthesina fullo* (Thunberg) (Hemiptera: Heteroptera)

**DOI:** 10.1371/journal.pone.0125970

**Published:** 2015-05-08

**Authors:** Yang Liu, Haoyang Wu, Qiang Xie, Wenjun Bu

**Affiliations:** Institute of Entomology, College of Life Sciences, Nankai University, Tianjin, China; Institute of Vegetables and Flowers, Chinese Academy of Agricultural Science, CHINA

## Abstract

*Erthesina fullo* (Thunberg, 1783) is an economically important heteropteran species in China. Since only three nucleotide sequences of this species (COI, 16S rRNA, and 18S rRNA) appear in the GenBank database so far, no analysis of the molecular mechanisms underlying *E*. *fullo*’s resistance to insecticide and environmental stress has been accomplished. We reported a *de novo* assembled and annotated transcriptome for adult *E*. *fullo* using the Illumina sequence system. A total of 53,359,458 clean reads of 4.8 billion nucleotides (nt) were assembled into 27,488 unigenes with an average length of 750 bp, of which 17,743 (64.55%) were annotated. In the present study, we identified 88 putative cytochrome P450 sequences and analyzed the evolution of cytochrome P450 superfamilies, genes of the CYP3 clan related to metabolizing xenobiotics and plant natural compounds, in *E*. *fullo*, increasing the candidate genes for the molecular mechanisms of insecticide resistance in P450. The sequenced transcriptome greatly expands the available genomic information and could allow a better understanding of the mechanisms of insecticide resistance at the systems biology level.

## Introduction

To understand the diversification and evolution of life, especially for diversified radiated insects, more transcriptomic data for non-model organisms is crucial. This is a serious situation for the Heteroptera group, which has no important model organism. The transcriptomic data of very few species has been reported [1–5]. With the amazing development of high-throughput sequencing technology and sharp declines of the cost per species, transcriptomic analysis is becoming a possible and efficient way to achieve large-scale genetic data of insect genome with high heterozygosity at low cost. The transcriptome of *Erthesina*. *fullo* will be critical for further genome sequencing and annotation, as well as the inherent defense mechanisms of traditional control and resistance development mechanisms.

*Erthesina fullo* (Thunberg, 1783), the yellow marmorated stink bug, is one of the most widely distributed phytophagous pests in East Asia and has caused severe loss to many commercially important fruits, such as apples, cherries, and pears [6, 7]. In addition to an agricultural pest, *E*. *fullo* may invade home and indoor spaces as adults before reappearance in spring according to the observation in the lab. It is well-known that the brown marmorated stink bug (BMSB, *Halyomorpha halys*) is an invasive insect native to Asia and has poses a considerable ecological and economical threat to North America and Europe [8]. According to the research and investigation [6, 7], the occurrence regularity and plant hosts of *E*. *fullo* are quite similar with those of *H*. *halys*. Given proper condition and circumstances, *E*. *fullo* would constitute an ecological and economic danger to the world as a representative of a group of agriculturally significant true bugs. However, only three nucleotide sequences (COI, 16S rRNA, and 18S rRNA) were found in the GenBank database.

The cytochrome P450 enzymes, or CYP genes, are one of the largest gene families, which have corresponding representatives in almost all extant lineages, from bacteria to animals [9]. Their important tasks include the metabolism of alien chemicals of natural or synthetic origin which make them quite important in the research of insecticide resistance mechanisms to control devastating economic pests [10]. Like other detoxifying enzymes, the diversified gene families, by which the cytochrome P450 superfamily of monooxygenases are encoded, are difficult to entirely characterize. Phylogenetic analysis of insect P450s protein sequences revealed that they are distributed into four major clades [11] which are strongly supported by bootstrap analysis (CYP2, CYP3, CYP4 and Mitochondrial CYP clans). Nevertheless, most of the related research on P450 focuses on the model organisms or the world-wide pests [12, 13]. The diversified constitution and function of the non-model organisms or local pests remains mysterious.

Current resistance mechanism research in insects posits that insecticide resistance commonly comes up through two chief mechanisms, mutating target site of certain insecticide to reduce their mutual binding [14] (e.g. the voltage-gated sodium channel for pyrethroids) or enhancing the metabolism or sequestration of insecticide by functional enzymes such as cytochrome P450 monooxygenases, carboxyl/choline esterases (CCEs) and glutathione-S-transferases (GSTs) [15–20]. In the case of P450s for example, CYP6G1 was linked to insecticide resistance in DDT-resistant *Drosophila melanogaster* Meigen (Diptera: Drosophilidae) [21], and CYP6Z1 in the mosquito malaria vector *Anopheles gambiae* Giles (Diptera: Culicidae) was capable of directly metabolizing DDT [22]. However, due to insufficient genomic information and the complexity of detoxification gene families and pathways involved in resistance, the cytochrome P450-induced insecticide resistant mechanism is not well understood and pest control efforts are therefore hampered.

Here, we used the Illumina sequencing system to generate a *de novo* assembled and annotated transcriptome for adult *E*. *fullo*, a species that has lacked genomic resources. We also identified and analyzed the evolution of cytochrome P450 superfamilies using the Maximum Likelihood method and Bayesian inference of likelihood. The outcome of this study remarkably increase the genome-level information and will potentially contribute to improving our understanding of this pest at the systems biology level.

## Materials and Methods

### *De Novo* Transcriptome Sequencing and Assembly

Adult specimens of *E*. *fullo* were collected from a peer tree in an orchard of Xiping Village (32°13.322N, 110°59.346E), Fang County, Shiyan City, Hubei Province, China, on June 12, 2013. This location did not require a specific permission for the field studies, and our study did not involve endangered or protected species either. After wild collection, fresh tissue was stored in liquid nitrogen to prevent total RNA from degradation. After RNA extraction and enrichment, cDNA library construction and library normalization, the transcriptome was sequenced with the Illumina Hiseq 2000 system, which is widely employed for large-scale transcriptome data acquisition. After data acquisition, we used FastQC (http://www.bioinformatics.babraham.ac.uk/projects/fastqc) to assess the quality of the transcriptomic data. An unpublished integrative program based on Bioconductor was utilized to conduct the read cleaning including multiple N (stands for unknown nucleotide) base, low quality region on both ends (quality score less than Q20), possible mixed adaptor and contaminated rRNA and virus sequences. After quality assessment and read cleaning, reads are assembled using Trinity [23].

### Bioinformatic Analysis

Unigene function annotation was screened against the NCBI Non-redundant protein sequences (Nr), SwissProt, Kyoto Encyclopedia of Genes and Genomes (KEGG), and Cluster of Orthologous Groups of proteins (COG) protein databases using BLASTX with a cutoff E-value of 10^–5^. Unigene Gene Ontology (GO) classification was executed with Blast2GO using default parameters. The Clusters of Orthologous Groups of proteins (COG) were a framework for functional transcriptome or genome analysis based on homology, which were delineated by comparing protein sequences encoded in representative complete genomes of major phylogenetic lineages. Unigene GO classification was executed with the Java-based software Blast2GO [24–27] using default parameters. The protein coding region prediction of unigenes that had no hits when searching against protein databases (ordered by priority: Nr, SwissProt, KEGG and COG) was executed with ESTScan [28] while metabolic pathway analysis was processed with the KEGG annotation system.

### Gene Family Evolution

We aligned amino acid sequences using MAFFT v. 7.130 [29] for each mutually found orthologous gene. Refinement was executed with MUSCLE v. 3.8.31 [30, 31] for each gene. The cytochrome P450 clustering was inferred using the Maximum Likelihood method and Bayesian inference of likelihood, based on the best model given by ProtTest 3.4 [32] with the Akaike Information Criterion (AIC) method, the Bayesian information criterion (BIC) and the hierarchical likelihood-ratio tests [33]. The analysis involved 203 amino acid sequences and a total of 806 positions in the final data matrix, 88 of which belonged to *E*. *fullo* while the others came from other well-explored taxons. Evolutionary analyses were conducted in RAxML v8.0 [34] with 1000 rapid bootstrap replicates and checked a posteriori if the computation of bootstrap trees was sufficient using the bootstrapping criteria (default settings) [35]. Bayesian inference of likelihood was perfomed in a modified GPU-accelerated version of MrBayes [36] with aamodelpr = mixed. The analysis was proceeded until the effective sample size (ESS) was larger than 200 or the standard deviation of split frequencies was below 0.01. Two simultaneous runs of 5,000,000 generations were conducted while each set was sampled every 100 generations with a burn-in of 20,000. The posterior probabilities (PP) of all the clades were computed from the remaining trees.

### Data Submission

The clean data generated in this study have been deposited at the National Center for Biotechnology Information (NCBI) with the following characters: BioProject (accession ID PRJNA263698), BioSample (accession number SAMN03105778), and Short Read Archive (SRA) under accession code SRP048862.

## Results and Discussion

### Sequencing and Basic Sequence Assembly

A normalized library of adult *E*. *fullo* was sequenced with the Illumina Hiseq 2000 system, which generated 53,359,458 clean reads and a total of 4,802,351,220 nucleotides (nt). Reads were assembled into 50,378 contigs with an average length of 378 bp using Trinity [23]. After mapping back to contigs and extending, we obtained 27,488 unigenes with a mean length of 750 bp and N50 value of 1185 bp owing to the help of pair-end reads (Table 1, S1 Fig).

**Table 1:** Overview of the *E*. *fullo* transcriptome.

Analyzed item	Detailed number
Total Raw Reads	62,051,164
Total Clean Reads	53,359,458
Total Clean Nucleotides (nt)	4,802,351,220
Assembled Unigenes	27,488
Annotated Unigenes	17,743
Unigenes with reasonable protein coding region	19,486

### Unigene Function Annotation

**Fig 1:**
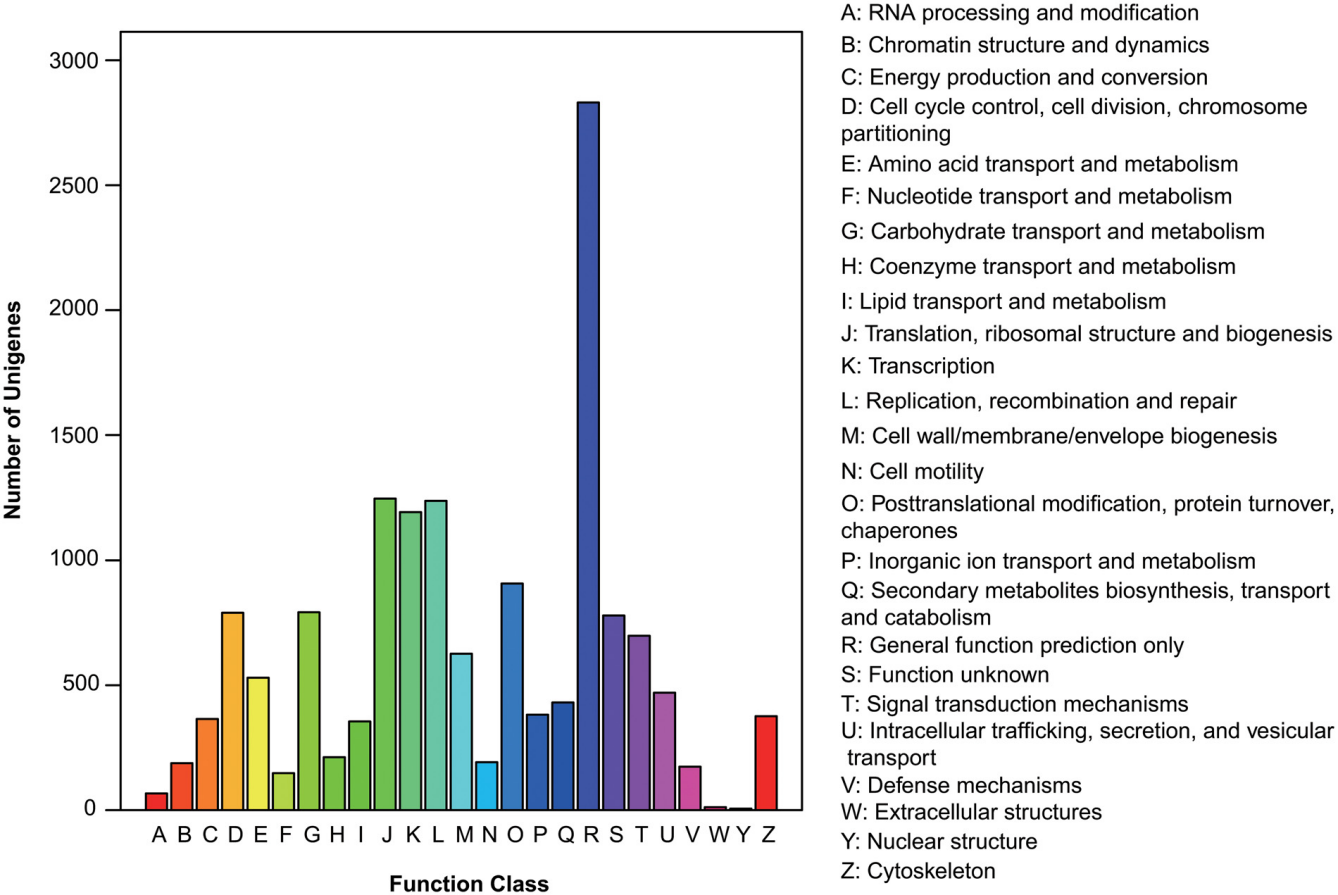
COG function classification of *E*. *fullo* unigene sequences. The Clusters of Orthologous Groups of proteins (COG) were a framework for functional transcriptome or genome analysis based on compared protein sequences encoded in representative complete genomes. A total of 6994 unigenes were grouped into COG function classifications.

**Fig 2:**
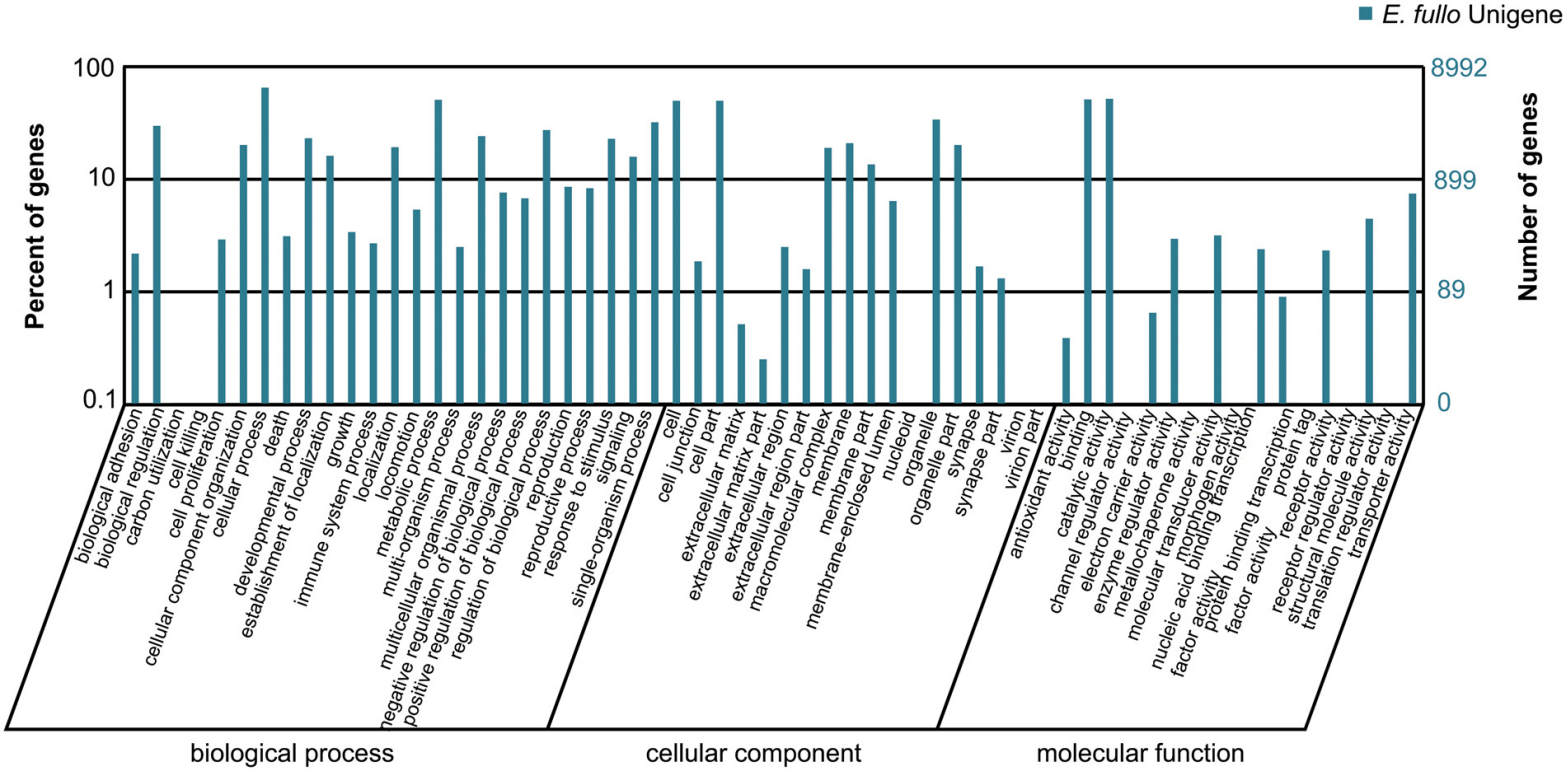
GO Classification for the transcriptome of *E*. *fullo*. Gene Ontology (GO) covers three domains: cellular component, molecular function and biological process. In total, 8992 unigenes can be classified into certain functional groups.

According to annotation results against the Nr database, a total of 17,276 distinct sequences (62.85% of all assembled unigenes) were matched to known proteins. Of the matched known proteins, 52.9% had strong similarity with common insects such as *Tribolium castaneum* (Herbst) (Coleoptera: Tenebrionidae) and *Acyrthosiphon pisum* (Harris) (Hemiptera: Aphididae) (S2 Fig). Due to the insufficiency of heteropteran genome reference, Nr annotation of species distribution is not as efficient as re-sequencing species with a known genome. The COG database was constructed by mutual comparison of protein coding genes in every complete genome. The principal is that each COG should consist of individual proteins or groups of paralogs from at least 3 lineages. When considering proteins from a certain genome or transcriptome, the comparison will lead to the best match functional group of proteins (Fig 1). For GO classification, unigenes were divided into three ontologies, which are cellular component, molecular function and biological process. 8,992 unigenes (32.71% of entirety) were categorized into different functional groups in 257 KEGG pathways. Cellular process and catalytic activity were the two largest ontologies, comprising 5,703 and 4,533 unigenes severally (Fig 2).

### Unigene Metabolic Pathway Analysis and Protein Coding Region Prediction

First, unigene sequences were searched against protein databases using BLASTX with a cutoff E-value of 10^–5^ in the following order: Nr, SwissProt, KEGG and COG. Unigene sequences that had hits in a preferential database were not moved to the next round. Then the relatively best blast results information was adopted to extract a Coding DNA Sequence (CDS) from unigene sequences. Unigenes with no hits in blast results were predicted by the coding region detection program ESTScan [28, 37, 38]. A total number of 19,486 unigenes had reasonable protein coding regions.

KEGG annotation system was utilized to conduct the Unigene metabolic pathway analysis. 12,804 unigenes were mapped to 257 KEGG pathways; metabolic pathways contained 1,800 unigenes (14.06%), substantially larger than any other pathways involved, followed by regulation of actin cytoskeleton (4.28%), focal adhesion (3.75%), and pathways in cancer (3.53%) (S1 Table).

### Cytochrome P450 and Insecticide Resistance

**Fig 3:**
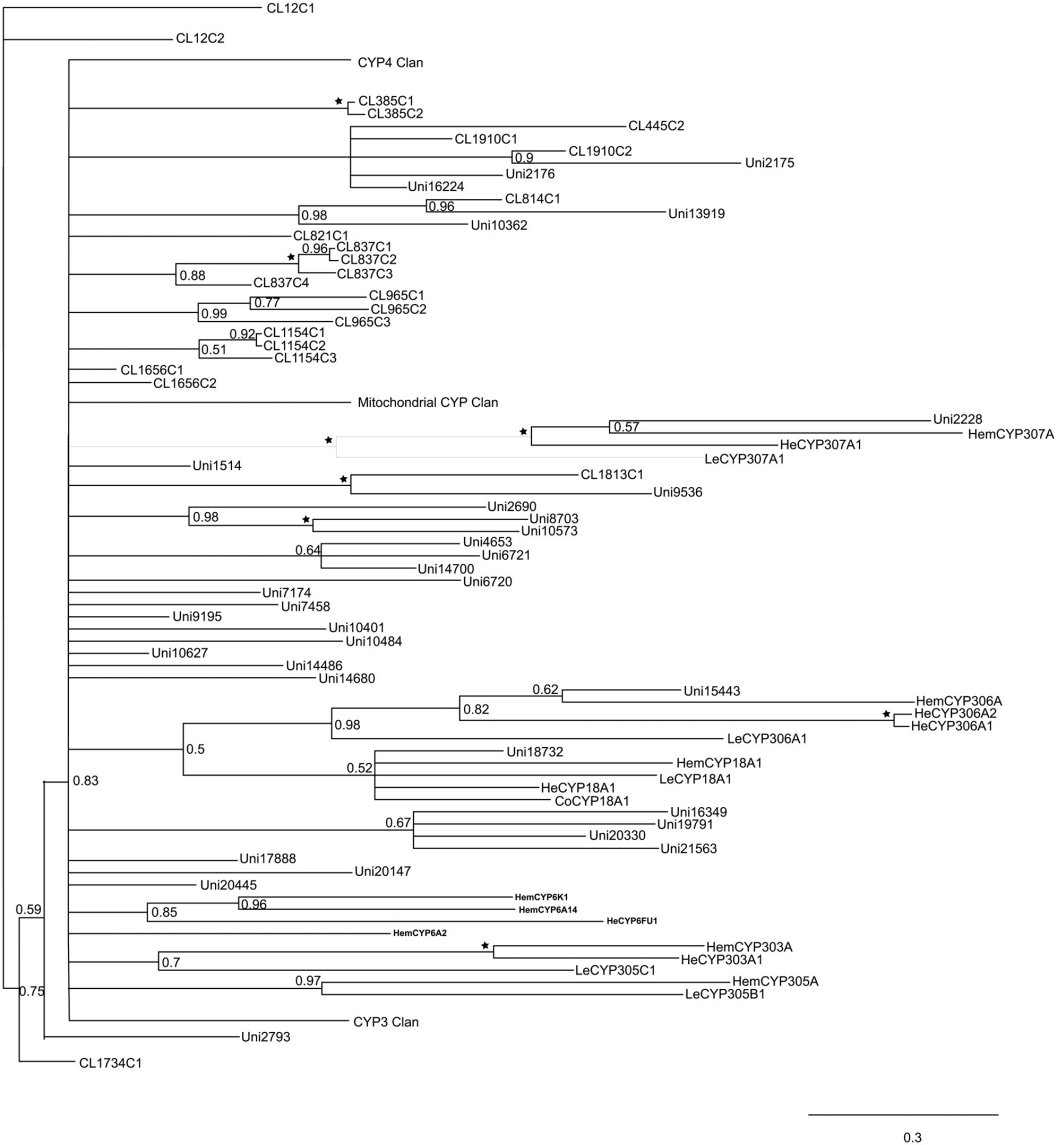
Phylogenetic analysis of *E*. *fullo* cytochrome P450s using MrBayes.

**Fig 4:**
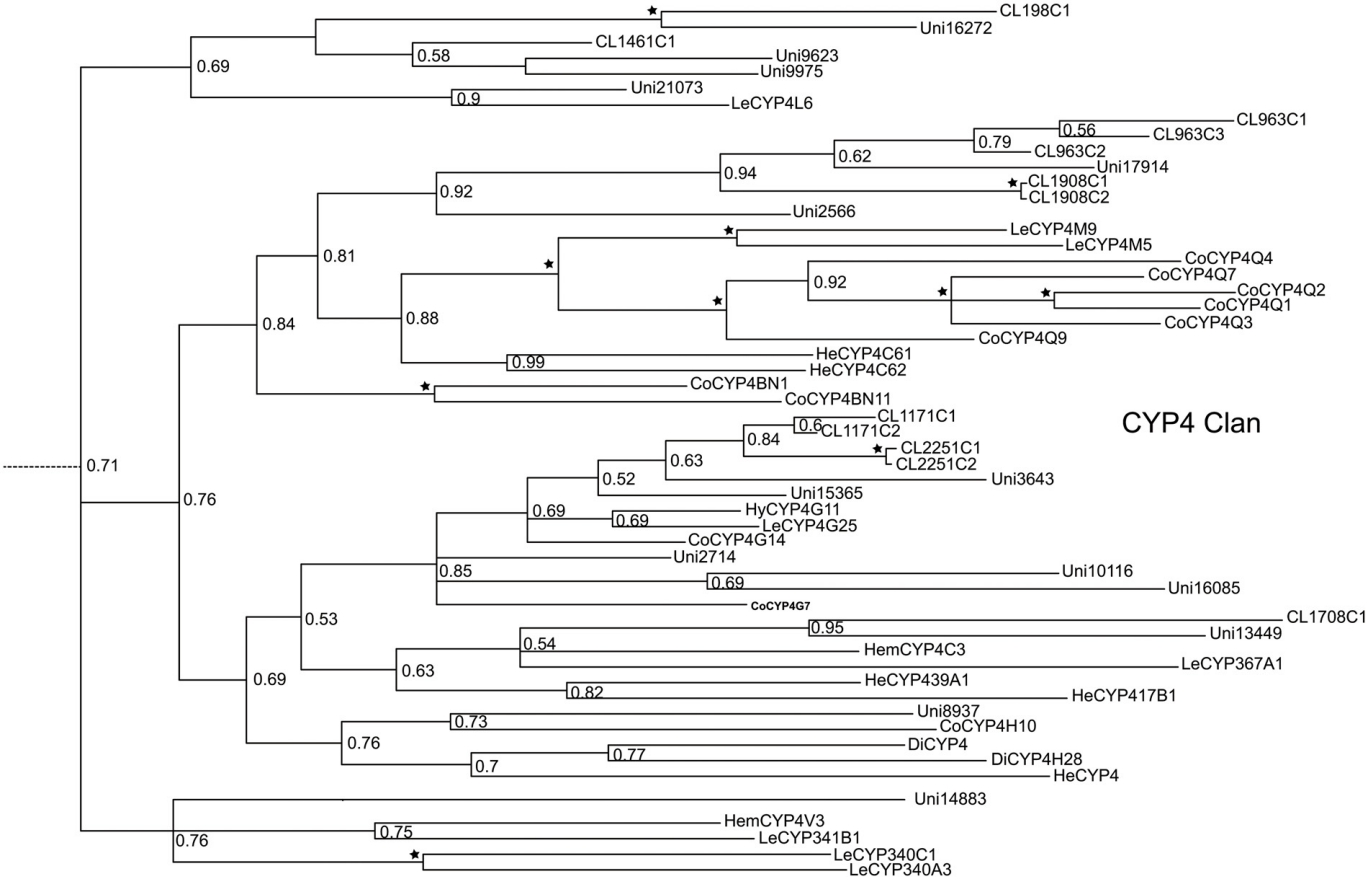
*E*. *fullo* cytochrome P450s CYP4 clan using MrBayes. A total of 26 unigenes were classified into the CYP4 clan by phylogenetic analysis.

**Fig 5:**
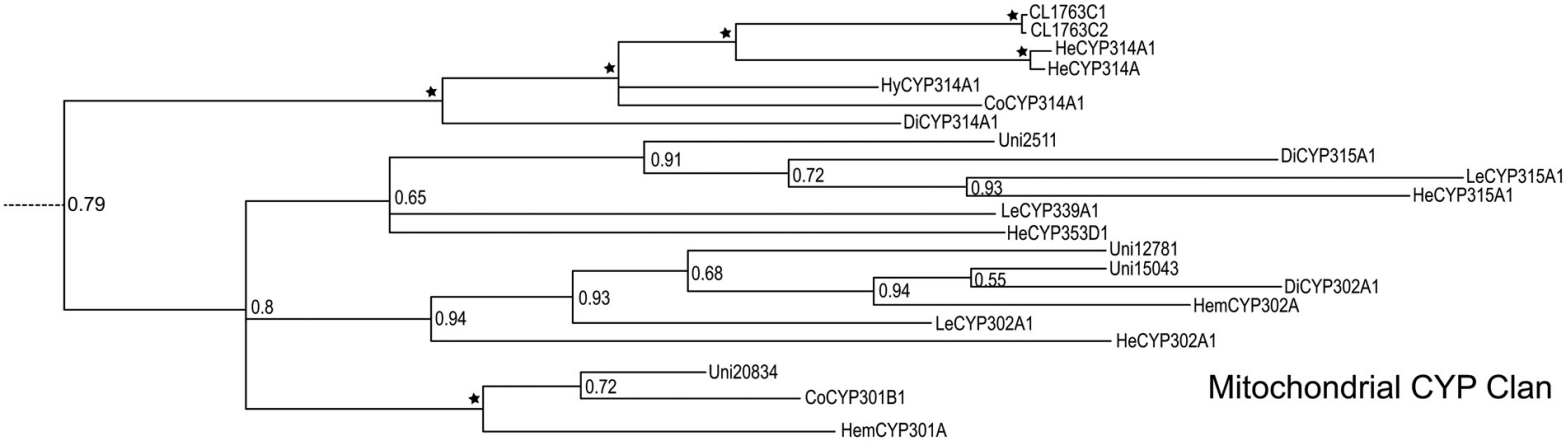
*E*. *fullo* cytochrome P450s mitochondrial CYP clan using MrBayes. Only 6 unigenes were sorted into the mitochondrial CYP clan.

**Fig 6:**
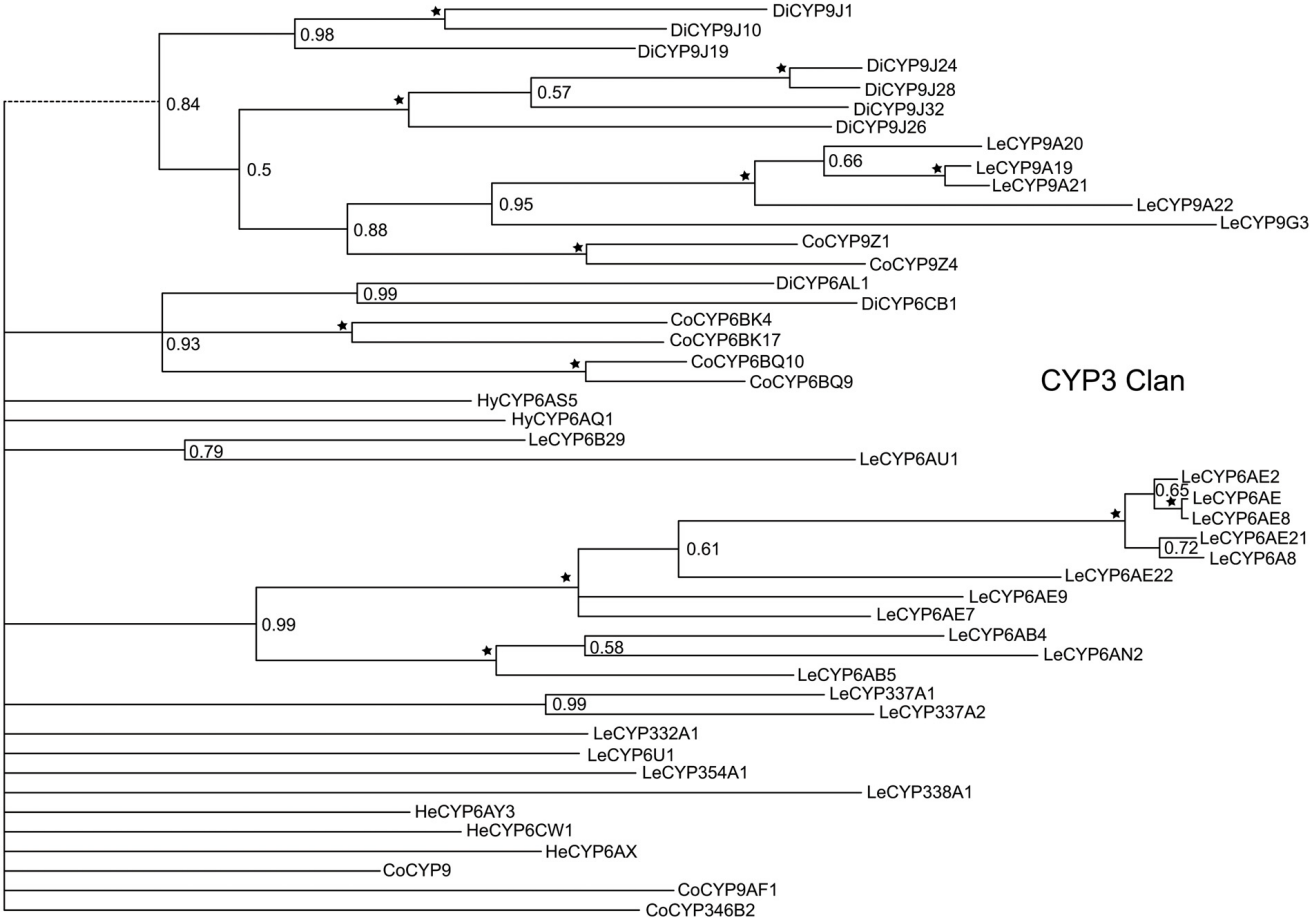
*E*. *fullo* cytochrome P450s CYP3 clan using MrBayes. In total, 45 unigenes were categorized into the CYP3 clan. Branches labeled by pentagrams represent PP = 1.00. The first two or three characters of the sequence name stands for the orders the sequences belong to (Hem: *A*. *pisum*, Di: *A*. *aegypti*, Hy: *A*. *mellifera*, Le: *B*. *mori*, He: *L*. *striatella*, Co: *T*. *castaneum*).

**Fig 7:**
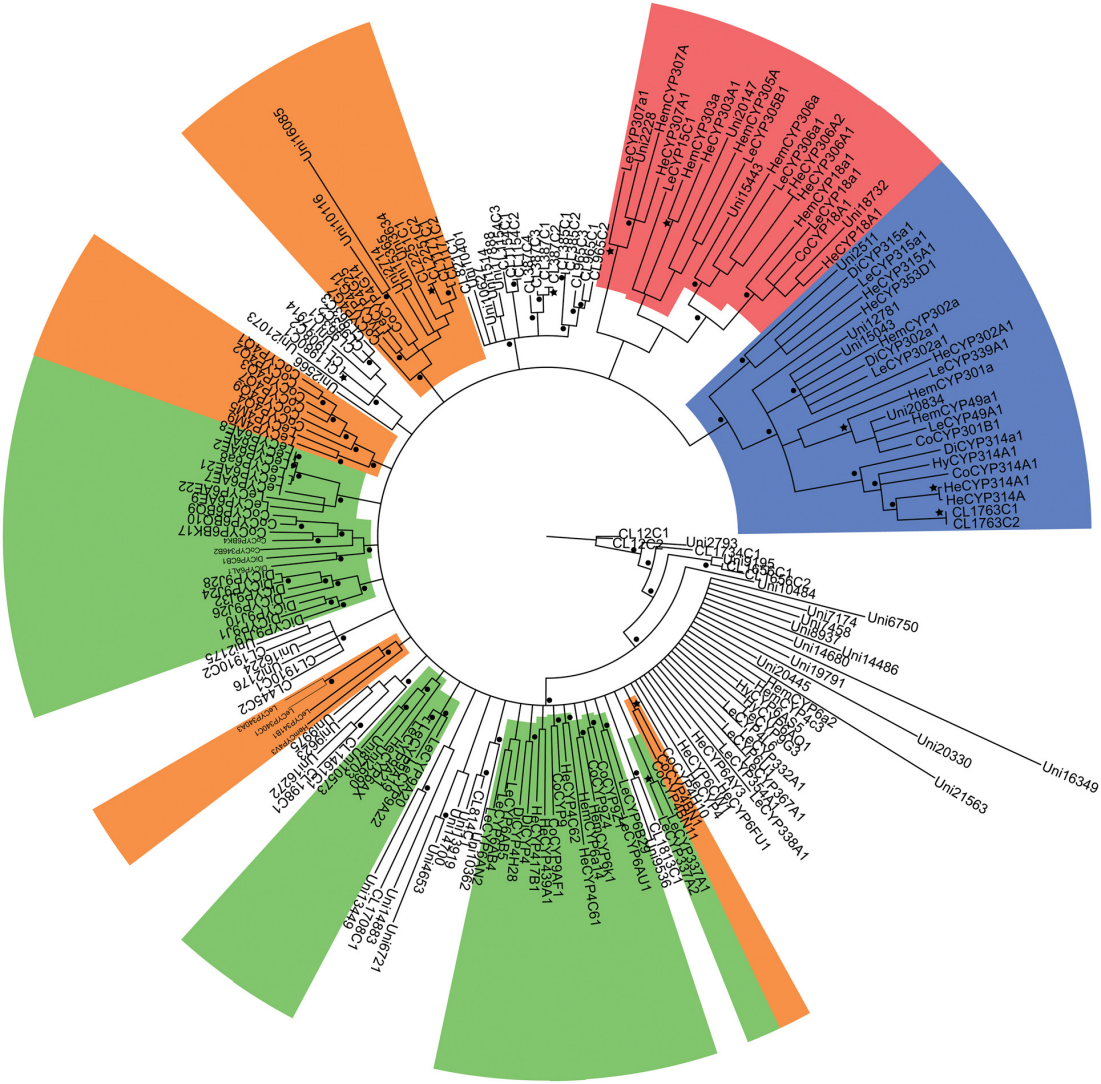
Phylogenetic analysis of *E*. *fullo* cytochrome P450s using RAxML. Sequences with orange shadowing belong to the CYP4 clan while those with blue shading were classified into the mitochondrial CYP clan Green shadowing indicates the CYP3 clan and red background represents the CYP2 clan. Branches labeled by pentagrams represent bootstrap value = 1.00; clades tagged by spots had bootstrap values greater than 0.70.

A total of 88 possible P450 sequences were found in the assembled transcriptome. All of them were screened against the Nr database using BLASTX with a cutoff E-value of 10^–5^ to specifically identify to certain gene families. Meanwhile, the identified sequences and related P450 genes from *Acrythosiphon pisum* (Harris) (Hemiptera: Aphididae), *Aedes aegypti* (Linnaeus) (Diptera: Culicidae), *Apis mellifera* (Linnaeus) (Hymenoptera: Apidae), *Bombyx mori* (Linnaeus) (Lepidoptera: Bombycidae), *Laodelphax striatella* (Fallén) (Hemiptera: Delphacidae) and *Tribolium castaneum* (Herbst) (Coleoptera: Tenebrionidae) were analyzed with Maximum Likelihood method and Bayesian inference of likelihood, which classified most of the genes into the CYP2, CYP3, CYP4 and mitochondrial CYP clans (Figs 3–7). 26 of 88 possible sequences were classified as CYP4 clan members, while 6 and 3 of them were categorized as mitochondrial CYP and CYP2 clans respectively. Except for 4 undefined sequences, other possible clans had an equal phylogenetic position with members of the CYP3 clan (Table 2).For the Maximum Likelihood analysis, most of the relative relationship among four clans is as same as that of Bayesian analysis except for several members of CYP4 clan have merged into the CYP 3 clan as unresolved branches. Traditionally, the maximum likelihood analysis is widely used to reconstruct different level of phylogenetic relationship and supposed to be the most effective and reliable way. However, when the nucleotide substitutions broadly varies among different branches, incorrect topological structure might be treated as the actual tree more easily [39]. Concerning cytochrome P450 genes, the identity rules for family and subfamily designations are all members nominally over 40% and 55% identical separately [10]. The sequences are quite diversified compared to other genes involved in phylogenetic analysis. The high diversity and rapid evolution rate of P450 genes in different insects might be the reason for unresolved branches.

**Table 2:** Approximate Numbers of CYP genes in various insect species.

Species	CYP2 clan	Mitochondrial CYP clan	CYP3 clan	CYP4 clan	CYPome size
*Drosophila melanogaster*	6	11	36	32	85
*Anopheles gambiae*	10	9	42	45	106
*Aedes aegypti*	9	10	92	68	179
*Bombyx mori*	8	13	31	29	81
*Apis mellifera*	8	6	28	4	46
*Nasonia vitripennis*	7	7	47	29	89
*Tribolium castaneum*	8	9	70	44	131
*Acyrthosiphon pisum*	10	8	33	32	83
*Pediculus humanus*	8	8	12	9	37
*Myzus persicae*	3	1	63	48	115
*Trialeurodes vaporariorum*	3	7	34	13	57
*Bactrocera oleae*	2	13	28	17	60
*Nilaparvata lugens*	10	12	18	27	67
*Erthesina fullo*	3	6	49 (4 undefined)	26	88

Compared to the relative affinities in other Hemiptera whose P450 genes have been to some extent identified, more P450 genes in *E*. *fullo* were found in the CYP3 clan. Genes from the CYP3 clan were testified to have the ability of metabolizing xenobiotics and plant natural compounds [11], meanwile, they appear to share some common characteristics with the “environmental response genes” defined by Berenbaum [40]. The results implied that *E*. *fullo* could evolve rapidly in response to its environment, including a high level of insecticide resistance. Searching the KEGG pathway analysis, genes from the mitochondrial CYP and CYP2 clans belonged to the “Insect hormone biosynthesis” pathway (ko00981). Only 13 out of 88 possible P450 genes were classified into the “Metabolism of xenobiotics by cytochrome P450” (ko00980), “Drug metabolism-cytochrome P450” (ko00982) and “Drug metabolism-other enzymes” (ko00983) pathways, which show a probably vital and direct role in insecticide resistance, while the other P450 genes play a basic role in metabolic pathways. Further studies of the role of these genes in hemipteran detoxification mechanisms would benefit our understanding of the interaction between phytophagous insects and plants.

For arthropod pests, P450s are well known to play an important role in metabolic resistance to a series of major insecticide classes including organochlorides, organophosphates (Ops), carbamates, pyrethroids, neonicotinoids and insect growth regulators [41]. Generally, the chemical basis of P450s-induced insecticide resistance is the reinforcement of catalysis activity against insecticide metabolism performed at three levels: increasing transcriptional efficiency at the transcription level, the amplification of structural genes at the gene level and increasing the stability of mRNA or protein at the translation level. Nevertheless, no research on P450-induced insecticide resistance mechanisms of *E*. *fullo* was reported due to the lack of genomic or transcriptomic information. The rising amount of P450s in insects with insecticide resistance had been shown to a result of overexpressing through up-regulation via modificaiton in cis- or trans-acting regulatory loci [19]. However, it has now been implicated that there exist correlation between the duplication or amplification of P450s and the insecticide resistance in some insects such as *D*. *melanogaster*, *Culex quinquefasciatus* (Say) (Diptera: Culicidae), *Anopheles funestus* (Giles) (Diptera: Culicidae) and *Myzus persicae* (Sulzer) (Hemiptera: Aphididae) [42–45]. For hemipterans, amplification of genes that encode esterases has been reported [46]. In the gene amplification studies of arthropod pest, the delineation of the molecular basis for esterase-mediated resistance in the peach potato aphid, *M*. *persicae*, is one of the most successful and complete case [47, 48]. So far, no amplification of P450 genes has been reported in *E*. *fullo*. According to previous research, the P450 gene families involved in up-regulation and amplification are CYP4, CYP6 and CYP9 (belonging to the CYP3 and CYP4 clans), all of which were identified in *E*. *fullo*. Our transcriptome provides a set number of P450 genes for the further exploration of insecticide resistance mechanisms and the potential discovery of novel functional targets for insecticides. The transcriptome of *E*. *fullo* also detected dozens of esterase sequences with potential relevance to heteropteran insecticide resistance. Further comparison and analysis of esterase genes and gene copy numbers or folds between wild-type and resistance-phenotype bugs could explain the currently unknown insecticide resistance mechanisms of the true bugs. Furthermore, the availability of large-scale genomic and transcriptosomic data makes the discovery of more gene members in different detoxifying enzyme gene families easier.

## Conclusions

Using the Illumina sequence system, we reported a *de novo* assembled and annotated transcriptome for the phytophagous pest *E*. *fullo*. A total of 53,359,458 clean reads of 4.8 billion nucleotides (nt) were assembled into 27,488 unigenes with a mean length of 750 bp, from which 17,743 (64.55%) were annotated with different databases. In the present study, we also identified and analyzed the evolution of cytochrome P450 superfamilies. Genes of the CYP3 clan related to metabolizing xenobiotics and plant natural compounds were found in *E*. *fullo*, which increases the candidate genes for further research into the molecular mechanisms of P450 insecticide resistance. Moreover, more CYP3 clan genes were found in *E*. *fullo*, which implied that *E*. *fullo* could evolve rapidly in response to its environment, including a high level of insecticide resistance. Our study greatly expands the available genomic information and especially provides a better understanding of the mechanisms of insecticide resistance at the systems biology level for this economically important non-model organism.

## Supporting Information

S1 Fig Unigene Lengths Distribution in the transcriptome of *E*. *fullo*. (EPS)

S2 Fig Distribution statistics of Unigene Annotation against the BLAST Nr database. (a) The distribution of BLAST hits E-value for each unigene with a cutoff E-value of 10^–5^ when searching against the Nr database. (b) Distribution of the sequence similarity of BLAST hits. (c) Species distribution of sequences belonging to model insects.
(EPS)

S1 Table Unigene Metabolic Pathway Analysis. (DOC)

## References

[1] Ribeiro JM, Genta FA, Sorgine MH, Logullo R, Mesquita RD, Paiva-Silva GO, et al An insight into the transcriptome of the digestive tract of the bloodsucking bug, *Rhodnius prolixus* . PLoS Negl Trop Dis. 2014; 8(1): e2594 10.1371/journal.pntd.0002594 24416461 PMC3886914

[2] Zou D, Coudron TA, Liu C, Zhang L, Wang M, Chen H. Nutrigenomics in *Arma chinensis*: transcriptome analysis of *Arma chinensis* fed on artificial diet and Chinese oak silk moth *Antheraea pernyi* pupae. PLoS One. 2013; 8(4): e60881 10.1371/journal.pone.0060881 23593338 PMC3623872

[3] Zhu F, Gujar H, Gordon JR, Haynes KF, Potter MF, Palli SR. Bed bugs evolved unique adaptive strategy to resist pyrethroid insecticides. Sci Rep. 2013; 3: 1456 10.1038/srep01456 23492626 PMC3596983

[4] Ribeiro JM, Assumpção TC, Pham VM, Francischetti IM, Reisenman CE. An insight into the sialotranscriptome of *Triatoma rubida* (Hemiptera: Heteroptera). J Med Entomol. 2012; 49(3): 563–572. 22679863 10.1603/me11243 PMC3544468

[5] Bai X, Mamidala P, Rajarapu SP, Jones SC, Mittapalli O. Transcriptomics of the bed bug (*Cimex lectularius*). PLoS One. 2011; 6(1): e16336 10.1371/journal.pone.0016336 21283830 PMC3023805

[6] Li Q, Cheng A, Wang H, Zhang W. The prevention and control of the brown marmorated sting bug and yellow marmorated sting bug. Plant Doctor. 1998; 11(1): 17–18.

[7] Zhang Y, Zhao D, Liu W, Wang K. The occurrence regularity and control of major true bugs pests in orchard. Modern rural technology. 2010; 13: 23–24. 10.1002/yd.371 21240949

[8] Sparks ME, Shelby KS, Kuhar D, Gundersen-Rindal DE. Transcriptome of the Invasive Brown Marmorated Stink Bug, *Halyomorpha halys* (Stål) (Heteroptera: Pentatomidae). PLoS One. 2014; 9(11): e111646 10.1371/journal.pone.0111646 25386688 PMC4227672

[9] Werck-Reichhart D, Feyereisen R. Cytochromes P450: A success story. Genome Biol. 2000; 1(6): 3003.1–3003.9. 10.1186/gb-2000-1-6-reviews3003 PMC138896 11178272

[10] Feyereisen R. Evolution of insect P450. Biochem Soc Trans. 2006; 34(Pt 6): 1252–1255. 17073796 10.1042/BST0341252

[11] Feyereisen R. Insect CYP genes and P450 enzymes In: Gilbert LI, editors. Insect Molecular Biology and Biochemistry. London: Elsevier / Academic Press; 2012 pp. 236–316.

[12] You M, Yue Z, He W, Yang X, Yang G, Xie M, et al A heterozygous moth genome provides insights into herbivory and detoxification. Nat Genet. 2013; 45(2): 220–225. 10.1038/ng.2524 23313953

[13] Xue J, Zhou X, Zhang C, Yu L, Fan H, Wang Z, et al Genomes of the rice pest brown planthopper and its endosymbionts reveal complex complementary contributions for host adaptation. Genome Biol. 2014; 15(12): 521 10.1186/s13059-014-0521-0 25609551 PMC4269174

[14] Pittendrigh BR, Margam VM, Sun L, Huesing JE. Resistance in the postgenomics age In: Onstad DW, editors. Insect Resistance Management: Biology, Economics and Prediction. USA: Elsevier; 2008 pp. 39–68.

[15] Ranson H, Claudianos C, Ortelli F, Abgrall C, Hemingway J, Sharakhova MV, et al Evolution of supergene families associated with insecticide resistance. Science. 2002; 298(5591): 179–181. 12364796 10.1126/science.1076781

[16] Feyereisen R. Insect cytochrome P450 In: Gilbert LI, Iatrou K, Gill SS, editors. Comprehensive Molecular Insect Science—Biochemistry and Molecular Biology. Amsterdam: Elsevier; 2005 pp. 1–77.

[17] Oakeshott JG, Claudianos C, Campbell PM, Newcomb RD, Russell RJ. Biochemical genetics and genomics of insect esterases In: Gilbert LI, Iatrou K, Gill SS, editors. Comprehensive Molecular Insect Science-Pharmacology. Amsterdam: Elsevier; 2005 pp. 309–381.

[18] Ranson H, Hemingway J. Glutathione transferases In: Gilbert LI, Iatrou K, Gill SS, editors. Comprehensive Molecular Insect Science-Pharmacology. Amsterdam: Elsevier; 2005 pp. 383–402.

[19] Li X, Schuler MA, Berenbaum MR. Molecular mechanisms of metabolic resistance to synthetic and natural xenobiotics. Annu Rev Entomol. 2007; 52: 231–253. 16925478 10.1146/annurev.ento.51.110104.151104

[20] Daane KM, Johnson MW. Olive fruit fly: Managing an ancient pest in modern times. Annu Rev Entomol. 2010; 55: 151–169. 10.1146/annurev.ento.54.110807.090553 19961328

[21] Jones RT, Bakker SE, Stone D, Shuttleworth SN, Boundy S, McCart C, et al Homology modelling of Drosophila cytochrome P450 enzymes associated with insecticide resistance. Pest Manag Sci. 2010; 66(10): 1106–1115. 10.1002/ps.1986 20583201

[22] Kasai S, Komagata O, Itokawa K, Shono T, Ng LC, Kobayashi M, et al Mechanisms of pyrethroid resistance in the dengue mosquito vector, *Aedes aegypti*: target site insensitivity, penetration, and metabolism. PLoS Negl Trop Dis. 2014; 8(6): e2948 10.1371/journal.pntd.0002948 24945250 PMC4063723

[23] Grabherr MG, Haas BJ, Yassour M, Levin JZ, Thompson DA, Amit I, et al Full-length transcriptome assembly from RNA-seq data without a reference genome. Nat Biotechnol. 2011; 29(7): 644–652. 10.1038/nbt.1883 21572440 PMC3571712

[24] Conesa A, Götz S, García-Gómez JM, Terol J, Talón M, Robles M. Blast2GO: a universal tool for annotation, visualization and analysis in functional genomics research. Bioinformatics. 2005; 21(18): 3674–3676. 16081474 10.1093/bioinformatics/bti610

[25] Conesa A, Götz S. Blast2GO: A Comprehensive Suite for Functional Analysis in Plant Genomics. Int J Plant Genomics. 2008; 2008: 619832 10.1155/2008/619832 18483572 PMC2375974

[26] Götz S, García-Gómez JM, Terol J, Williams TD, Nagaraj SH, Nueda MJ, et al High-throughput functional annotation and data mining with the Blast2GO suite. Nucleic Acids Res. 2008; 36(10): 3420–3435. 10.1093/nar/gkn176 18445632 PMC2425479

[27] Götz S, Arnold R, Sebastián-León P, Martín-Rodríguez S, Tischler P, Jehl MA, et al B2G-FAR, a species centered GO annotation repository. Bioinformatics. 2011; 27(7): 919–924. 10.1093/bioinformatics/btr059 21335611 PMC3065692

[28] Iseli C, Jongeneel CV, Bucher P. ESTScan: a program for detecting, evaluating, and reconstructing potential coding regions in EST sequences. Proc Int Conf Intell Syst Mol Biol. 1999; 1999: 138–148. 10786296

[29] Katoh K, Standley DM. MAFFT multiple sequence alignment software version 7: improvements in performance and usability. Mol Biol Evol. 2013; 30(4): 772–780. 10.1093/molbev/mst010 23329690 PMC3603318

[30] Edgar RC. MUSCLE: multiple sequence alignment with high accuracy and high throughput. Nucleic Acids Res. 2004; 32(5): 1792–1797. 15034147 10.1093/nar/gkh340 PMC390337

[31] Edgar RC. MUSCLE: a multiple sequence alignment method with reduced time and space complexity. BMC Bioinformatics. 2004; 5: 113 15318951 10.1186/1471-2105-5-113 PMC517706

[32] Darriba D, Taboada GL, Doallo R, Posada D. ProtTest 3: fast selection of best-fit models of protein evolution. Bioinformatics. 2011; 27(8): 1164–1165. 10.1093/bioinformatics/btr088 21335321 PMC5215816

[33] Guindon S, Gascuel O. A simple, fast, and accurate algorithm to estimate large phylogenies by maximum likelihood. Syst Biol. 2003; 52(5): 696–704. 14530136 10.1080/10635150390235520

[34] Stamatakis A. RaxML Version 8: A tool for Phylogenetic Analysis and Post-Analysis of Large Phylogenies. Bioinformatics. 2014; 30(9): 1312–1313. 10.1093/bioinformatics/btu033 24451623 PMC3998144

[35] Pattengale ND, Alipour M, Bininda-Emonds OR, Moret BM, Stamatakis A. How many bootstrap replicates are necessary? J Comput Biol. 2010; 17(3): 337–354. 10.1089/cmb.2009.0179 20377449

[36] Bao J, Xia H, Zhou J, Liu X, Wang G. Efficient Implementation of MrBayes on multi-GPU. Mol Biol Evol. 2013; 30(6): 1471–1479. 10.1093/molbev/mst043 23493260 PMC3649675

[37] Lottaz C, Iseli C, Jongeneel CV, Bucher P. Modeling sequencing errors by combining Hidden Markov models. Bioinformatics. 2003; 19 Suppl: II 103–112. 14534179 10.1093/bioinformatics/btg1067

[38] Wasmuth JD, Blaxter ML. prot4EST: Translating Expressed Sequence Tags from neglected genomes. BMC Bioinformatics. 2004; 5: 187 15571632 10.1186/1471-2105-5-187 PMC543579

[39] Yang Z. Computational Molecular Evolution (Oxford Series in Ecology and Evolution). New York: Oxford University Press; 2006 pp. 102–146.

[40] Berenbaum MR. Postgenomic chemical ecology: From genetic code to ecological interactions. J Chem Ecol. 2002; 28(5): 873–896. 12049229 10.1023/a:1015260931034

[41] Schuler MA. P450s in plant-insect interactions. Biochim Biophys Acta. 2011; 1814(1): 36–45. 10.1016/j.bbapap.2010.09.012 20883828

[42] Daborn PJ, Yen JL, Bogwitz MR, Le Goff G, Feil E, Jeffers S, et al A single p450 allele associated with insecticide resistance in Drosophila. Science. 2002; 297(5590): 2253–2256. 12351787 10.1126/science.1074170

[43] Itokawa K, Komagata O, Kasai S, Okamura Y, Masada M, Tomita T. Genomic structures of Cyp9m10 in pyrethroid resistant and susceptible strains of *Culex quinquefasciatus* . Insect Biochem Mol Biol. 2010; 40(9): 631–640. 10.1016/j.ibmb.2010.06.001 20600899

[44] Wondji CS, Irving H, Morgan J, Lobo NF, Collins FH, Hunt RH, et al Two duplicated P450 genes are associated with pyrethroid resistance in *Anopheles funestus*, a major malaria vector. Genome Res. 2009; 19(3): 452–459. 10.1101/gr.087916.108 19196725 PMC2661802

[45] Puinean AM, Foster SP, Oliphant L, Denholm I, Field LM, Millar NS, et al Amplification of a cytochrome P450 gene is associated with resistance to neonicotinoid insecticides in the aphid *Myzus persicae* . PloS Genet. 2010; 6(6): e1000999 10.1371/journal.pgen.1000999 20585623 PMC2891718

[46] Bass C, Field LM. Gene amplification and insecticide resistance. Pest Manag Sci. 2011; 67(8): 886–890. 10.1002/ps.2189 21538802

[47] Devonshire AL, Field LM, Foster SP, Moores GD, Williamson MS, Blackman RL. The evolution of insecticide resistance in the peach potato aphid, *Myzus persicae* . Philos Trans R Soc Lond B Biol Sci. 1998; 353: 1677–1684.

[48] Bass C, Puinean AM, Zimmer CT, Denholm I, Field LM, Foster SP, et al The evolution of insecticide resistance in the peach potato aphid, *Myzus persicae* . Insect Biochem Mol Biol. 2014; 51: 41–51. 10.1016/j.ibmb.2014.05.003 24855024

