# Supplementary material for: Novel Detection of Insecticide Resistance Related P450 Genes and Transcriptome Analysis of the Hemimetabolous Pest Erthesina fullo (Thunberg) (Hemiptera: Heteroptera)
Source: PLoS One. 2015 May 8;10(5):e0125970. doi: 10.1371/journal.pone.0125970 (PMC4425472; doi:10.1371/journal.pone.0125970)
Supplement: S1 Table — (DOC) [file pone.0125970.s003.doc]

S1 Table. Unigene Metabolic Pathway Analysis

| No. | Pathway | All genes with pathway annotation (12804) | Pathway ID |
| --- | --- | --- | --- |
| 1 | [Metabolic pathways](#gene1) | 1800(14.06%) | ko01100 |
| 2 | [Regulation of actin cytoskeleton](#gene2) | 548(4.28%) | ko04810 |
| 3 | [Focal adhesion](#gene3) | 480(3.75%) | ko04510 |
| 4 | [Pathways in cancer](#gene4) | 452(3.53%) | ko05200 |
| 5 | [RNA transport](#gene5) | 386(3.01%) | ko03013 |
| 6 | [Purine metabolism](#gene6) | 347(2.71%) | ko00230 |
| 7 | [Vascular smooth muscle contraction](#gene7) | 346(2.7%) | ko04270 |
| 8 | [Spliceosome](#gene8) | 331(2.59%) | ko03040 |
| 9 | [Endocytosis](#gene9) | 325(2.54%) | ko04144 |
| 10 | [Tight junction](#gene10) | 322(2.51%) | ko04530 |
| 11 | [HTLV-I infection](#gene11) | 316(2.47%) | ko05166 |
| 12 | [Epstein-Barr virus infection](#gene12) | 307(2.4%) | ko05169 |
| 13 | [Huntington's disease](#gene13) | 298(2.33%) | ko05016 |
| 14 | [Calcium signaling pathway](#gene14) | 283(2.21%) | ko04020 |
| 15 | [Lysosome](#gene15) | 277(2.16%) | ko04142 |
| 16 | [Protein processing in endoplasmic reticulum](#gene16) | 269(2.1%) | ko04141 |
| 17 | [Phagosome](#gene17) | 265(2.07%) | ko04145 |
| 18 | [Ubiquitin mediated proteolysis](#gene18) | 263(2.05%) | ko04120 |
| 19 | [Dilated cardiomyopathy](#gene19) | 262(2.05%) | ko05414 |
| 20 | [Hypertrophic cardiomyopathy (HCM)](#gene20) | 251(1.96%) | ko05410 |
| 21 | [Salmonella infection](#gene21) | 250(1.95%) | ko05132 |
| 22 | [MAPK signaling pathway](#gene22) | 247(1.93%) | ko04010 |
| 23 | [Alzheimer's disease](#gene23) | 246(1.92%) | ko05010 |
| 24 | [mRNA surveillance pathway](#gene24) | 244(1.91%) | ko03015 |
| 25 | [Influenza A](#gene25) | 243(1.9%) | ko05164 |
| 26 | [Pyrimidine metabolism](#gene26) | 241(1.88%) | ko00240 |
| 27 | [Transcriptional misregulation in cancer](#gene27) | 238(1.86%) | ko05202 |
| 28 | [Insulin signaling pathway](#gene28) | 236(1.84%) | ko04910 |
| 29 | [Chemokine signaling pathway](#gene29) | 233(1.82%) | ko04062 |
| 30 | [Cell cycle](#gene30) | 230(1.8%) | ko04110 |
| 31 | [Herpes simplex infection](#gene31) | 229(1.79%) | ko05168 |
| 32 | [Bile secretion](#gene32) | 227(1.77%) | ko04976 |
| 33 | [Amoebiasis](#gene33) | 223(1.74%) | ko05146 |
| 34 | [Wnt signaling pathway](#gene34) | 220(1.72%) | ko04310 |
| 35 | [Adherens junction](#gene35) | 209(1.63%) | ko04520 |
| 36 | [Gastric acid secretion](#gene36) | 207(1.62%) | ko04971 |
| 37 | [Ribosome biogenesis in eukaryotes](#gene37) | 198(1.55%) | ko03008 |
| 38 | [Axon guidance](#gene38) | 197(1.54%) | ko04360 |
| 39 | [Leukocyte transendothelial migration](#gene39) | 193(1.51%) | ko04670 |
| 40 | [Pathogenic Escherichia coli infection](#gene40) | 192(1.5%) | ko05130 |
| 41 | [Tuberculosis](#gene41) | 191(1.49%) | ko05152 |
| 42 | [Lysine degradation](#gene42) | 190(1.48%) | ko00310 |
| 43 | [Ribosome](#gene43) | 189(1.48%) | ko03010 |
| 44 | [Vibrio cholerae infection](#gene44) | 182(1.42%) | ko05110 |
| 45 | [Cardiac muscle contraction](#gene45) | 182(1.42%) | ko04260 |
| 46 | [Oocyte meiosis](#gene46) | 176(1.37%) | ko04114 |
| 47 | [Phosphatidylinositol signaling system](#gene47) | 173(1.35%) | ko04070 |
| 48 | [ABC transporters](#gene48) | 170(1.33%) | ko02010 |
| 49 | [Bacterial invasion of epithelial cells](#gene49) | 169(1.32%) | ko05100 |
| 50 | [Parkinson's disease](#gene50) | 166(1.3%) | ko05012 |
| 51 | [Fc gamma R-mediated phagocytosis](#gene51) | 165(1.29%) | ko04666 |
| 52 | [Neurotrophin signaling pathway](#gene52) | 165(1.29%) | ko04722 |
| 53 | [Pancreatic secretion](#gene53) | 165(1.29%) | ko04972 |
| 54 | [Peroxisome](#gene54) | 164(1.28%) | ko04146 |
| 55 | [Oxidative phosphorylation](#gene55) | 163(1.27%) | ko00190 |
| 56 | [Viral myocarditis](#gene56) | 160(1.25%) | ko05416 |
| 57 | [ECM-receptor interaction](#gene57) | 153(1.19%) | ko04512 |
| 58 | [Neuroactive ligand-receptor interaction](#gene58) | 149(1.16%) | ko04080 |
| 59 | [Prostate cancer](#gene59) | 148(1.16%) | ko05215 |
| 60 | [Dopaminergic synapse](#gene60) | 148(1.16%) | ko04728 |
| 61 | [T cell receptor signaling pathway](#gene61) | 147(1.15%) | ko04660 |
| 62 | [Toxoplasmosis](#gene62) | 140(1.09%) | ko05145 |
| 63 | [RNA polymerase](#gene63) | 140(1.09%) | ko03020 |
| 64 | [Shigellosis](#gene64) | 139(1.09%) | ko05131 |
| 65 | [RNA degradation](#gene65) | 138(1.08%) | ko03018 |
| 66 | [Dorso-ventral axis formation](#gene66) | 133(1.04%) | ko04320 |
| 67 | [Progesterone-mediated oocyte maturation](#gene67) | 133(1.04%) | ko04914 |
| 68 | [Glycerophospholipid metabolism](#gene68) | 130(1.02%) | ko00564 |
| 69 | [GnRH signaling pathway](#gene69) | 130(1.02%) | ko04912 |
| 70 | [Protein digestion and absorption](#gene70) | 129(1.01%) | ko04974 |
| 71 | [ErbB signaling pathway](#gene71) | 128(1%) | ko04012 |
| 72 | [Inositol phosphate metabolism](#gene72) | 127(0.99%) | ko00562 |
| 73 | [Drug metabolism - other enzymes](#gene73) | 126(0.98%) | ko00983 |
| 74 | [Melanogenesis](#gene74) | 126(0.98%) | ko04916 |
| 75 | [Hepatitis C](#gene75) | 123(0.96%) | ko05160 |
| 76 | [Salivary secretion](#gene76) | 122(0.95%) | ko04970 |
| 77 | [Small cell lung cancer](#gene77) | 121(0.95%) | ko05222 |
| 78 | [Measles](#gene78) | 119(0.93%) | ko05162 |
| 79 | [Starch and sucrose metabolism](#gene79) | 117(0.91%) | ko00500 |
| 80 | [Amyotrophic lateral sclerosis (ALS)](#gene80) | 115(0.9%) | ko05014 |
| 81 | [Alcoholism](#gene81) | 111(0.87%) | ko05034 |
| 82 | [Aminoacyl-tRNA biosynthesis](#gene82) | 111(0.87%) | ko00970 |
| 83 | [Morphine addiction](#gene83) | 110(0.86%) | ko05032 |
| 84 | [Glutamatergic synapse](#gene84) | 109(0.85%) | ko04724 |
| 85 | [Glycerolipid metabolism](#gene85) | 107(0.84%) | ko00561 |
| 86 | [Vitamin digestion and absorption](#gene86) | 106(0.83%) | ko04977 |
| 87 | [Basal transcription factors](#gene87) | 105(0.82%) | ko03022 |
| 88 | [TGF-beta signaling pathway](#gene88) | 105(0.82%) | ko04350 |
| 89 | [Retrograde endocannabinoid signaling](#gene89) | 103(0.8%) | ko04723 |
| 90 | [Long-term potentiation](#gene90) | 103(0.8%) | ko04720 |
| 91 | [Gap junction](#gene91) | 102(0.8%) | ko04540 |
| 92 | [Cholinergic synapse](#gene92) | 101(0.79%) | ko04725 |
| 93 | [Metabolism of xenobiotics by cytochrome P450](#gene93) | 101(0.79%) | ko00980 |
| 94 | [Antigen processing and presentation](#gene94) | 101(0.79%) | ko04612 |
| 95 | [Pentose and glucuronate interconversions](#gene95) | 100(0.78%) | ko00040 |
| 96 | [Drug metabolism - cytochrome P450](#gene96) | 99(0.77%) | ko00982 |
| 97 | [Cytosolic DNA-sensing pathway](#gene97) | 97(0.76%) | ko04623 |
| 98 | [Vasopressin-regulated water reabsorption](#gene98) | 97(0.76%) | ko04962 |
| 99 | [Retinol metabolism](#gene99) | 96(0.75%) | ko00830 |
| 100 | [GABAergic synapse](#gene100) | 96(0.75%) | ko04727 |
| 101 | [VEGF signaling pathway](#gene101) | 96(0.75%) | ko04370 |
| 102 | [Arrhythmogenic right ventricular cardiomyopathy (ARVC)](#gene102) | 96(0.75%) | ko05412 |
| 103 | [PPAR signaling pathway](#gene103) | 94(0.73%) | ko03320 |
| 104 | [Cell adhesion molecules (CAMs)](#gene104) | 94(0.73%) | ko04514 |
| 105 | [Hedgehog signaling pathway](#gene105) | 93(0.73%) | ko04340 |
| 106 | [Glutathione metabolism](#gene106) | 93(0.73%) | ko00480 |
| 107 | [Renal cell carcinoma](#gene107) | 88(0.69%) | ko05211 |
| 108 | [Amino sugar and nucleotide sugar metabolism](#gene108) | 88(0.69%) | ko00520 |
| 109 | [Fat digestion and absorption](#gene109) | 88(0.69%) | ko04975 |
| 110 | [Nucleotide excision repair](#gene110) | 87(0.68%) | ko03420 |
| 111 | [Fanconi anemia pathway](#gene111) | 86(0.67%) | ko03460 |
| 112 | [Colorectal cancer](#gene112) | 85(0.66%) | ko05210 |
| 113 | [Epithelial cell signaling in Helicobacter pylori infection](#gene113) | 85(0.66%) | ko05120 |
| 114 | [Phototransduction - fly](#gene114) | 85(0.66%) | ko04745 |
| 115 | [Pyruvate metabolism](#gene115) | 84(0.66%) | ko00620 |
| 116 | [Glycine, serine and threonine metabolism](#gene116) | 84(0.66%) | ko00260 |
| 117 | [Glioma](#gene117) | 84(0.66%) | ko05214 |
| 118 | [Chronic myeloid leukemia](#gene118) | 83(0.65%) | ko05220 |
| 119 | [Glycolysis / Gluconeogenesis](#gene119) | 82(0.64%) | ko00010 |
| 120 | [Jak-STAT signaling pathway](#gene120) | 82(0.64%) | ko04630 |
| 121 | [Notch signaling pathway](#gene121) | 82(0.64%) | ko04330 |
| 122 | [Porphyrin and chlorophyll metabolism](#gene122) | 81(0.63%) | ko00860 |
| 123 | [Arginine and proline metabolism](#gene123) | 81(0.63%) | ko00330 |
| 124 | [Steroid hormone biosynthesis](#gene124) | 79(0.62%) | ko00140 |
| 125 | [Fc epsilon RI signaling pathway](#gene125) | 78(0.61%) | ko04664 |
| 126 | [DNA replication](#gene126) | 78(0.61%) | ko03030 |
| 127 | [Rheumatoid arthritis](#gene127) | 78(0.61%) | ko05323 |
| 128 | [p53 signaling pathway](#gene128) | 77(0.6%) | ko04115 |
| 129 | [Endometrial cancer](#gene129) | 77(0.6%) | ko05213 |
| 130 | [B cell receptor signaling pathway](#gene130) | 76(0.59%) | ko04662 |
| 131 | [Synaptic vesicle cycle](#gene131) | 75(0.59%) | ko04721 |
| 132 | [Galactose metabolism](#gene132) | 75(0.59%) | ko00052 |
| 133 | [Valine, leucine and isoleucine degradation](#gene133) | 75(0.59%) | ko00280 |
| 134 | [Amphetamine addiction](#gene134) | 75(0.59%) | ko05031 |
| 135 | [Legionellosis](#gene135) | 75(0.59%) | ko05134 |
| 136 | [Other types of O-glycan biosynthesis](#gene136) | 73(0.57%) | ko00514 |
| 137 | [Prion diseases](#gene137) | 73(0.57%) | ko05020 |
| 138 | [mTOR signaling pathway](#gene138) | 72(0.56%) | ko04150 |
| 139 | [Basal cell carcinoma](#gene139) | 72(0.56%) | ko05217 |
| 140 | [Mineral absorption](#gene140) | 71(0.55%) | ko04978 |
| 141 | [Chagas disease (American trypanosomiasis)](#gene141) | 71(0.55%) | ko05142 |
| 142 | [Pertussis](#gene142) | 71(0.55%) | ko05133 |
| 143 | [Serotonergic synapse](#gene143) | 71(0.55%) | ko04726 |
| 144 | [Insect hormone biosynthesis](#gene144) | 70(0.55%) | ko00981 |
| 145 | [Osteoclast differentiation](#gene145) | 70(0.55%) | ko04380 |
| 146 | [NF-kappa B signaling pathway](#gene146) | 70(0.55%) | ko04064 |
| 147 | [Natural killer cell mediated cytotoxicity](#gene147) | 68(0.53%) | ko04650 |
| 148 | [Endocrine and other factor-regulated calcium reabsorption](#gene148) | 68(0.53%) | ko04961 |
| 149 | [Fructose and mannose metabolism](#gene149) | 68(0.53%) | ko00051 |
| 150 | [Pancreatic cancer](#gene150) | 68(0.53%) | ko05212 |
| 151 | [Toll-like receptor signaling pathway](#gene151) | 67(0.52%) | ko04620 |
| 152 | [Ascorbate and aldarate metabolism](#gene152) | 67(0.52%) | ko00053 |
| 153 | [Complement and coagulation cascades](#gene153) | 67(0.52%) | ko04610 |
| 154 | [N-Glycan biosynthesis](#gene154) | 66(0.52%) | ko00510 |
| 155 | [Cysteine and methionine metabolism](#gene155) | 66(0.52%) | ko00270 |
| 156 | [Base excision repair](#gene156) | 65(0.51%) | ko03410 |
| 157 | [Carbohydrate digestion and absorption](#gene157) | 64(0.5%) | ko04973 |
| 158 | [Olfactory transduction](#gene158) | 63(0.49%) | ko04740 |
| 159 | [Non-small cell lung cancer](#gene159) | 61(0.48%) | ko05223 |
| 160 | [Sphingolipid metabolism](#gene160) | 60(0.47%) | ko00600 |
| 161 | [Tyrosine metabolism](#gene161) | 60(0.47%) | ko00350 |
| 162 | [Adipocytokine signaling pathway](#gene162) | 58(0.45%) | ko04920 |
| 163 | [Citrate cycle (TCA cycle)](#gene163) | 58(0.45%) | ko00020 |
| 164 | [Apoptosis](#gene164) | 55(0.43%) | ko04210 |
| 165 | [Systemic lupus erythematosus](#gene165) | 53(0.41%) | ko05322 |
| 166 | [NOD-like receptor signaling pathway](#gene166) | 53(0.41%) | ko04621 |
| 167 | [Staphylococcus aureus infection](#gene167) | 53(0.41%) | ko05150 |
| 168 | [Propanoate metabolism](#gene168) | 52(0.41%) | ko00640 |
| 169 | [Tryptophan metabolism](#gene169) | 52(0.41%) | ko00380 |
| 170 | [Pentose phosphate pathway](#gene170) | 52(0.41%) | ko00030 |
| 171 | [Fatty acid metabolism](#gene171) | 52(0.41%) | ko00071 |
| 172 | [Type II diabetes mellitus](#gene172) | 52(0.41%) | ko04930 |
| 173 | [Homologous recombination](#gene173) | 50(0.39%) | ko03440 |
| 174 | [Hematopoietic cell lineage](#gene174) | 49(0.38%) | ko04640 |
| 175 | [Arachidonic acid metabolism](#gene175) | 49(0.38%) | ko00590 |
| 176 | [Glycosaminoglycan biosynthesis - heparan sulfate](#gene176) | 49(0.38%) | ko00534 |
| 177 | [Long-term depression](#gene177) | 49(0.38%) | ko04730 |
| 178 | [Phototransduction](#gene178) | 48(0.37%) | ko04744 |
| 179 | [beta-Alanine metabolism](#gene179) | 46(0.36%) | ko00410 |
| 180 | [Circadian rhythm - fly](#gene180) | 46(0.36%) | ko04711 |
| 181 | [Protein export](#gene181) | 45(0.35%) | ko03060 |
| 182 | [Proteasome](#gene182) | 45(0.35%) | ko03050 |
| 183 | [Glycosylphosphatidylinositol(GPI)-anchor biosynthesis](#gene183) | 44(0.34%) | ko00563 |
| 184 | [Cytokine-cytokine receptor interaction](#gene184) | 44(0.34%) | ko04060 |
| 185 | [Leishmaniasis](#gene185) | 43(0.34%) | ko05140 |
| 186 | [Aldosterone-regulated sodium reabsorption](#gene186) | 43(0.34%) | ko04960 |
| 187 | [Mismatch repair](#gene187) | 43(0.34%) | ko03430 |
| 188 | [Melanoma](#gene188) | 42(0.33%) | ko05218 |
| 189 | [Thyroid cancer](#gene189) | 42(0.33%) | ko05216 |
| 190 | [Folate biosynthesis](#gene190) | 41(0.32%) | ko00790 |
| 191 | [Butanoate metabolism](#gene191) | 40(0.31%) | ko00650 |
| 192 | [Malaria](#gene192) | 39(0.3%) | ko05144 |
| 193 | [Glycosaminoglycan degradation](#gene193) | 39(0.3%) | ko00531 |
| 194 | [Nicotine addiction](#gene194) | 39(0.3%) | ko05033 |
| 195 | [Cocaine addiction](#gene195) | 37(0.29%) | ko05030 |
| 196 | [alpha-Linolenic acid metabolism](#gene196) | 37(0.29%) | ko00592 |
| 197 | [Alanine, aspartate and glutamate metabolism](#gene197) | 37(0.29%) | ko00250 |
| 198 | [Acute myeloid leukemia](#gene198) | 36(0.28%) | ko05221 |
| 199 | [Ether lipid metabolism](#gene199) | 36(0.28%) | ko00565 |
| 200 | [Fatty acid elongation](#gene200) | 36(0.28%) | ko00062 |
| 201 | [Nicotinate and nicotinamide metabolism](#gene201) | 36(0.28%) | ko00760 |
| 202 | [Circadian rhythm - mammal](#gene202) | 35(0.27%) | ko04710 |
| 203 | [Primary immunodeficiency](#gene203) | 35(0.27%) | ko05340 |
| 204 | [MAPK signaling pathway - fly](#gene204) | 35(0.27%) | ko04013 |
| 205 | [Biosynthesis of unsaturated fatty acids](#gene205) | 32(0.25%) | ko01040 |
| 206 | [Terpenoid backbone biosynthesis](#gene206) | 32(0.25%) | ko00900 |
| 207 | [Collecting duct acid secretion](#gene207) | 31(0.24%) | ko04966 |
| 208 | [Regulation of autophagy](#gene208) | 31(0.24%) | ko04140 |
| 209 | [Bladder cancer](#gene209) | 31(0.24%) | ko05219 |
| 210 | [Linoleic acid metabolism](#gene210) | 30(0.23%) | ko00591 |
| 211 | [Riboflavin metabolism](#gene211) | 30(0.23%) | ko00740 |
| 212 | [Glyoxylate and dicarboxylate metabolism](#gene212) | 30(0.23%) | ko00630 |
| 213 | [SNARE interactions in vesicular transport](#gene213) | 30(0.23%) | ko04130 |
| 214 | [Fatty acid biosynthesis](#gene214) | 29(0.23%) | ko00061 |
| 215 | [Selenocompound metabolism](#gene215) | 28(0.22%) | ko00450 |
| 216 | [Proximal tubule bicarbonate reclamation](#gene216) | 28(0.22%) | ko04964 |
| 217 | [Renin-angiotensin system](#gene217) | 28(0.22%) | ko04614 |
| 218 | [Phenylalanine metabolism](#gene218) | 27(0.21%) | ko00360 |
| 219 | [Taste transduction](#gene219) | 27(0.21%) | ko04742 |
| 220 | [Mucin type O-Glycan biosynthesis](#gene220) | 26(0.2%) | ko00512 |
| 221 | [Other glycan degradation](#gene221) | 26(0.2%) | ko00511 |
| 222 | [Histidine metabolism](#gene222) | 25(0.2%) | ko00340 |
| 223 | [Non-homologous end-joining](#gene223) | 25(0.2%) | ko03450 |
| 224 | [Pantothenate and CoA biosynthesis](#gene224) | 24(0.19%) | ko00770 |
| 225 | [RIG-I-like receptor signaling pathway](#gene225) | 23(0.18%) | ko04622 |
| 226 | [Glycosaminoglycan biosynthesis - chondroitin sulfate](#gene226) | 23(0.18%) | ko00532 |
| 227 | [One carbon pool by folate](#gene227) | 20(0.16%) | ko00670 |
| 228 | [Sulfur relay system](#gene228) | 19(0.15%) | ko04122 |
| 229 | [African trypanosomiasis](#gene229) | 19(0.15%) | ko05143 |
| 230 | [Ubiquinone and other terpenoid-quinone biosynthesis](#gene230) | 19(0.15%) | ko00130 |
| 231 | [Cyanoamino acid metabolism](#gene231) | 18(0.14%) | ko00460 |
| 232 | [Caffeine metabolism](#gene232) | 17(0.13%) | ko00232 |
| 233 | [Glycosphingolipid biosynthesis - globo series](#gene233) | 16(0.12%) | ko00603 |
| 234 | [Autoimmune thyroid disease](#gene234) | 16(0.12%) | ko05320 |
| 235 | [Steroid biosynthesis](#gene235) | 15(0.12%) | ko00100 |
| 236 | [Sulfur metabolism](#gene236) | 15(0.12%) | ko00920 |
| 237 | [Glycosaminoglycan biosynthesis - keratan sulfate](#gene237) | 12(0.09%) | ko00533 |
| 238 | [Taurine and hypotaurine metabolism](#gene238) | 12(0.09%) | ko00430 |
| 239 | [Glycosphingolipid biosynthesis - lacto and neolacto series](#gene239) | 12(0.09%) | ko00601 |
| 240 | [Type I diabetes mellitus](#gene240) | 12(0.09%) | ko04940 |
| 241 | [Valine, leucine and isoleucine biosynthesis](#gene241) | 11(0.09%) | ko00290 |
| 242 | [Glycosphingolipid biosynthesis - ganglio series](#gene242) | 10(0.08%) | ko00604 |
| 243 | [Synthesis and degradation of ketone bodies](#gene243) | 10(0.08%) | ko00072 |
| 244 | [Lipoic acid metabolism](#gene244) | 9(0.07%) | ko00785 |
| 245 | [Maturity onset diabetes of the young](#gene245) | 9(0.07%) | ko04950 |
| 246 | [Butirosin and neomycin biosynthesis](#gene246) | 9(0.07%) | ko00524 |
| 247 | [D-Arginine and D-ornithine metabolism](#gene247) | 8(0.06%) | ko00472 |
| 248 | [Vitamin B6 metabolism](#gene248) | 7(0.05%) | ko00750 |
| 249 | [Primary bile acid biosynthesis](#gene249) | 6(0.05%) | ko00120 |
| 250 | [Biotin metabolism](#gene250) | 6(0.05%) | ko00780 |
| 251 | [Phenylalanine, tyrosine and tryptophan biosynthesis](#gene251) | 5(0.04%) | ko00400 |
| 252 | [Thiamine metabolism](#gene252) | 4(0.03%) | ko00730 |
| 253 | [D-Glutamine and D-glutamate metabolism](#gene253) | 1(0.01%) | ko00471 |
| 254 | [Lysine biosynthesis](#gene254) | 1(0.01%) | ko00300 |
| 255 | [Allograft rejection](#gene255) | 1(0.01%) | ko05330 |
| 256 | [Asthma](#gene256) | 1(0.01%) | ko05310 |
| 257 | [Graft-versus-host disease](#gene257) | 1(0.01%) | ko05332 |
